# Changes of calcium channel characteristics and its relationship with pancreatic islet β cell parasecretion in rat born small for gestational age

**DOI:** 10.1186/1687-9856-2013-S1-O52

**Published:** 2013-10-03

**Authors:** Yan-Yan Jin, Li Liang

**Affiliations:** 1 Department of Endocrinology, Children’s Hospital Affiliated to Medical School of Zhejiang University, Hangzhou 310003

## Objective

The experiment aimed to investigate the effect of intrauterine malnourishment on pancreatic isletβcell.

## Method

The animal model of small for gestation in rat was made under matemal calorie restriction by 50% from d1 of gestation until term, while the control groups named AGA rats, were obtained from normal pregnant rats. Both of SGA and AGA newborn rats were fed normally till 4 weeks and 8 weeks. Single islet β cell isolation and pancreatic tissue-slice technique were used to obtain β cells in neonatal rats and adult rats, respectively. Using whole cell patch clamp to record the characteristics of β cell voltage dependent calcium channel (such as current, reversal potential, I-V curve, activation curve, inactivation curve) in both SGA and AGA groups. Using capacitance measurement induced by a sequence of sine wave stimulus to reflect the insulin secretion. In a word, study the tendency of calcium channel kinetics and secretion property by timing.

## Results

The membrane capacitance of SGA was smaller than AGA rat after excluding the influence of whole cell membrane capacitance deviation both in neonatal rats, 4 weeks and 8 weeks (P<0.05). Also, The membrane capacitance of 8 weeks SGA rat was less than 4 weeks SGA rat (P<0.05), while the Cm between 4 weeks and 8weeks AGA rat had no significant difference. Among the calcium channel characteristics, 1) neonatal rats: Compared with group AGA, SGA showed much lower value of the calcium current density (Id) and peak current density of I-V curve (P<0.05), which was perfectly consistent with the result that test from depolarization from -40mV to 0mV, but there was no statistical difference found in reversal potential (P>0.05). The same result was obtained in activation curve and inactivation curve of calcium channel, whose half activated voltage/half inactivated voltage V1/2 and slope factor k between SGA and AGA both showed no significant difference (P>0.05) ; 2) adult rats: there was no statistical difference found in all average calcium current density (Id), including the total calcium, L-type and T-type. No significant difference was observed in the peak current density of I-V curve in SGA and AGA (P>0.05). However, the reversal potential in SGA group was much lower than control (P<0.05). The two group had the same shape of activation curve and inactivation curve of calcium channel, and 50% activated voltage between SGA and AGA had no significant difference, but the calcium channel of SGA was easily to be closed, the inactivated voltage in SGA group was much higher than control (P<0.01).

## Conclusion

The effect of intrauterine malnourishment on pancreatic isletβcell last in postnatal life and the destroy of βcell tends to aggravate with time. Low calcium current density on β cell membrane devotes to the decrease of β cell secretion in SGA neonatal rat. However, the current of calcium channel returns to normal level in childhood. The kinetics of calcium channel is changed significantly, which may be the reason that intrauterine malnourishment lead to the decrease of insulin secretion in adult life.

